# Early exposure to radiofrequency electromagnetic fields at 1850 MHz affects auditory circuits in early postnatal mice

**DOI:** 10.1038/s41598-018-36868-1

**Published:** 2019-01-23

**Authors:** Ju Hwan Kim, Yang Hoon Huh, Jae-Hun Lee, Jae Yun Jung, Seung Cheol Ahn, Hak Rim Kim

**Affiliations:** 1 Department of Pharmacology, College of Medicine, Dankook University, Cheonan-si, Chungnam South Korea; 2 Electron Microscopy Research Center, Korea Basic Science Institute, Ochang, Chungbuk South Korea; 3 Department of Otolaryngology-Head & Neck Surgery, College of Medicine, Dankook University, Cheonan-si, Chungnam South Korea; 4 Department of Physiology, College of Medicine, Dankook University, Cheonan-si, Chungnam South Korea

## Abstract

In the present study, we measured the spontaneous post synaptic currents (sPSCs) at the post synaptic principle cells of the medial nucleus of the trapezoid body (MNTB) in early postnatal mice after exposure to 1850 MHz radiofrequency electromagnetic fields (RF-EMF). sPSC frequencies and amplitudes were significantly increased in the RF-EMF exposed group. Moreover, the number of synaptic vesicles in the calyx of Held was significantly increased in presynaptic nerve terminals. Following RF-EMF exposure, the number of docking synaptic vesicles in the active zone increased, thereby expanding the total length of the presynaptic active zone in the calyx of Held. These data suggest that the increased sPSCs are a result of greater synaptic vesicle release from presynaptic nerves. However, we found no morphological changes in the inner hair cell ribbon synapses. Further, there were no significant changes in the hearing threshold of the auditory brainstem response at postnatal day 15. Our results indicate that exposure to RF-EMF at an early postnatal stage might directly affect brainstem auditory circuits, but it does not seem to alter general sound perception.

## Introduction

With the widespread adoption of wireless communication devices in recent decades, there has been a growing public interest in the various biological effects of radiofrequency (RF) electromagnetic fields (EMFs) emitted by mobile phones. Various studies reported that exposure to RF-EMF could affect neurodevelopment^1^, the blood-brain barrier^2^, demyelination^3^, neurite outgrowth^4^, neurotransmitter release^5^, cognitive impairment^6^, and ultimately, behavior^1,3^. Additional cellular effects of RF-EMF exposure may involve changes in the regulation of the cell cycle^7,8^ and alterations of intracellular and molecular pathways, such as the extracellular signal-regulated kinase pathway^2^. It has also been reported that exposure to RF-EMF can induce changes in the central nerve system, including autophagy^9^, apoptosis^10^, and synaptic trafficking of synaptic vesicles^5^.

In children, exposure to RF-EMF has rapidly increased, and mobile phone usage is the most important determinant of the amount of RF-EMF exposure^11^. As mobile phone use usually requires close contact with the ear, possible effects on the auditory system are of particular concern. Recently, Sun *et al*.^12^ reported that exposure to extremely low frequency electromagnetic field (ELF-EMF) at the early postnatal period could facilitate endocytosis and post-tetanic potentiation. This was possibly mediated via enhanced expression of P/Q subtype calcium channels in the medial nucleus of the trapezoid body (MNTB), which is one of the main auditory circuit nuclei in the brainstem^12^. Another study reported an effect of EMF exposure on neural differentiation and outgrowth at the embryonic period^13^. These findings about the cellular changes in response to EMF exposure motivated our research into its possible effect on spontaneous post synaptic currents (sPSCs).

Because the MNTB forms part of the auditory brainstem, it plays an essential role in the localization of sound sources and in the encoding of temporal features of complex sounds^14^. Thus, any changes to MNTB neurons may also affect auditory functioning. Despite the reported effects of ELF-EMFs on MNTB neurons, a possible influence of RF-EMFs on auditory circuits has not been investigated yet. Therefore, we used an 1850 MHz RF-EMF to examine its effect on synaptic transmission in the cochlear nucleus-MNTB of young postnatal mice.

We used whole cell voltage clamp to measure sPSCs at post synaptic principal cells in the MNTB. Furthermore, we inspected the fine structure of the calyx of Held presynaptic nerve terminal using transmission electron microscopy (TEM) to evaluate morphological alterations of synaptic vesicles and the active zones. Any possible physiological or morphological changes in MNTB neurons were studied using histological analyses of the ribbon synapse in the inner hair cells and by determining the hearing threshold using the auditory brainstem response.

## Results

### RF-EMF increases the frequency and amplitude of sPSCs at post synaptic cells of the MNTB

**Figure 1:**
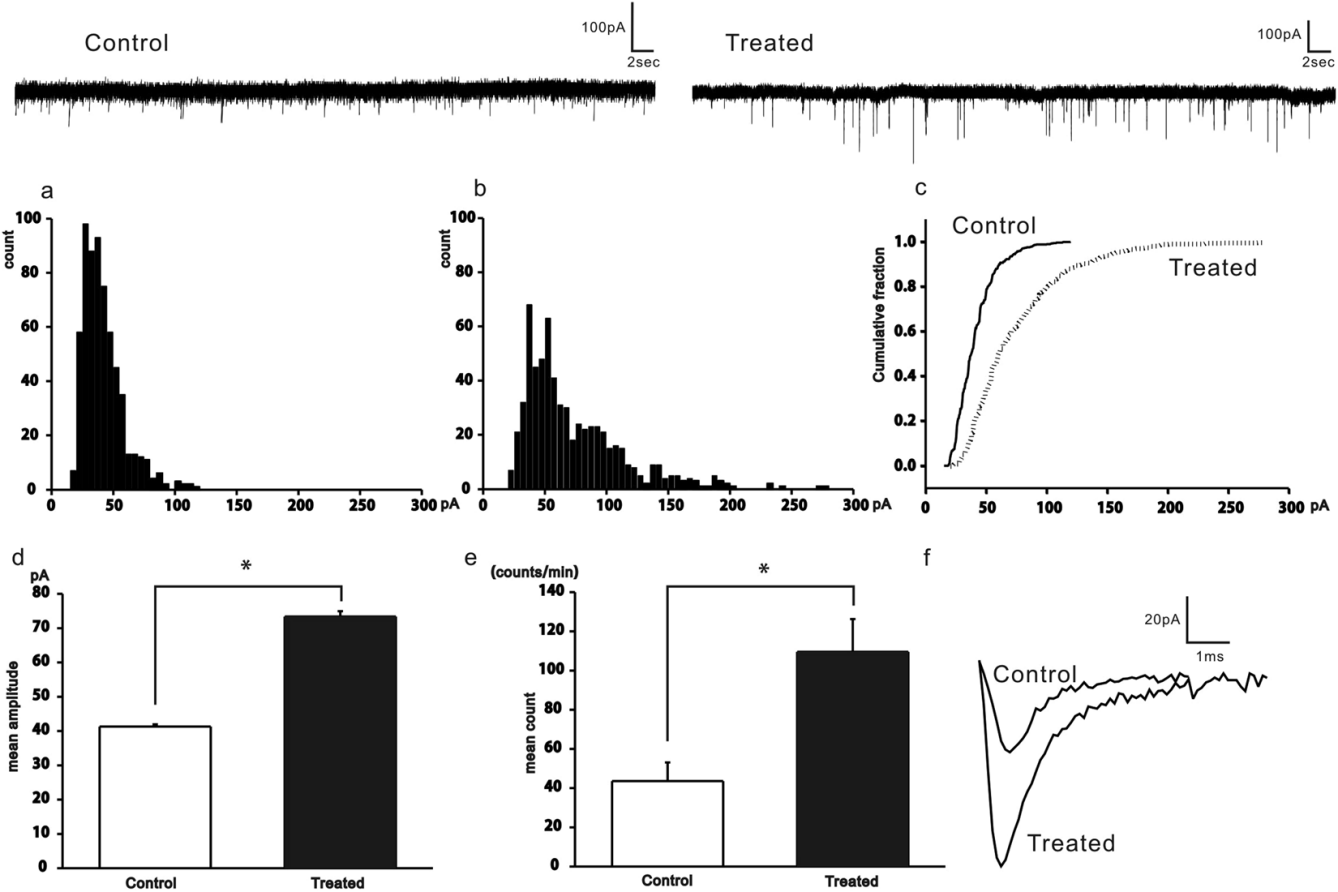
Frequencies and amplitudes of sPSCs were increased in the RF-EMF exposure group in the normal Ca^2+^ condition. Representative current traces are shown on top (left: control, right: RF-EMF exposed). Amplitude histograms are shown for the control (**a**) and RF-EMF exposed (**b**) groups. The cumulative fractions against amplitudes are shown in (**c**) (solid line: control, dashed line: RF-EMF exposed). The mean amplitudes and frequencies are shown in (**d**,**e**) respectively. The representative sPSCs (average of 37 sPSCs for control and 32 sPSCs for RF-EMF exposure group) are shown in (**f**). Statistical significance was evaluated using a two-tailed unpaired Student *t*-test. *P < 0.05.

To evaluate how RF-EMFs regulate synaptic transmission of MNTB synapses, sPSCs were recorded at post synaptic cells of the MNTB using the whole cell voltage clamp technique. At holding potential −60 mV, sPSCs were inhibited completely with the mixture of glutamate receptor blockers (10 μM D-APV and 5 μM CNQX) (data not shown), which indicated that sPSCs were glutamatergic. In the control group, the average frequency and amplitude at −60 mV were 43.6 ± 9.5 min^−1^ (n = 11) and 41.3 ± 0.7 pA (626 events from 11 cells), whereas they were 109.6 ± 16.7 min^−1^ (n = 14) and 73.4 ± 1.6 pA (647 events from 14 cells) in the RF-EMF exposed group (Fig. 1d,e). Two values were significantly different. The cumulative fraction plots confirm the different sPSC distributions between the two groups (Kolmogorov-Smirnov test, p < 0.5, Fig. 1c).

**Figure 2:**
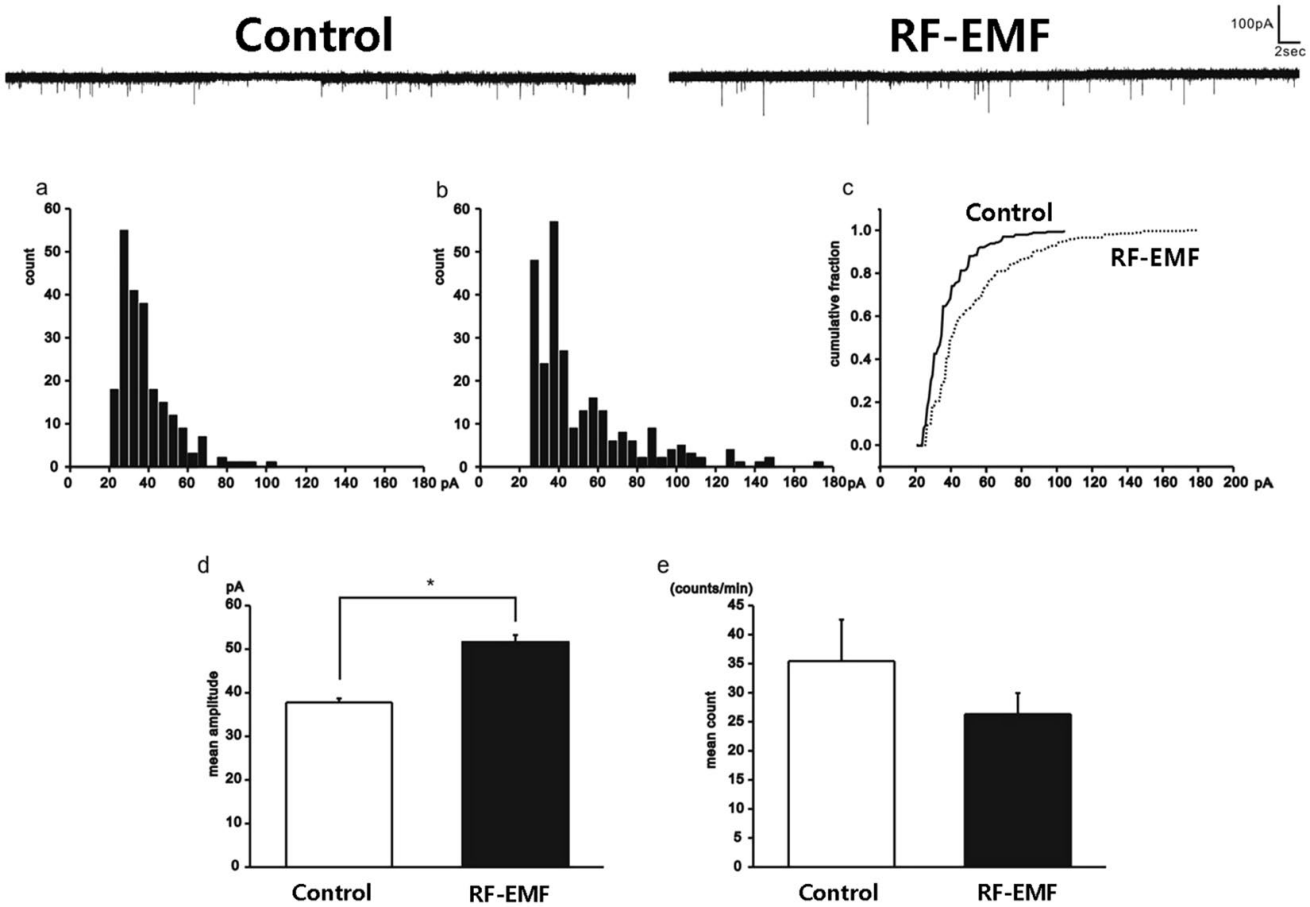
Only the amplitudes of sPSCs were increased in the RF-EMF exposure group with lower Ca^2+^ concentration. Representative current traces are shown above the graphs (left: control, right: RF-EMF exposed). Amplitude histograms are shown for the control (**a**) and RF-EMF exposed (**b**) groups. The cumulative fractions against amplitudes are shown in (**c**) (solid line: control, dashed line: RF-EMF exposed). The mean amplitudes and frequencies are shown in (**d**,**e**) respectively. Statistical significance was evaluated using a two-tailed unpaired Student *t*-test. *P < 0.05.

We repeated the experiment in a low calcium (reduced from 2 mM to 0.2 mM), high magnesium (increased from 1 mM to 4 mM) condition. The average frequency and amplitude at −60 mV were 35.5 ± 7.1 min^−1^ (n = 8) and 37.8 ± 0.9 pA (222 events from 8 cells) in the control group, compared to 26.3 ± 3.6 min^−1^ (n = 10) and 51.6 ± 1.6 pA (283 events from 10 cells) in RF-EMF exposed group (Fig. 2d,e). Only the difference in amplitudes was significant. Cumulative fraction plots also confirmed that the sPSC distributions differed between the two groups (Kolmogorov-Smirnov test, p < 0.5, Fig. 2c). These results suggest that neurotransmitter release at the presynaptic terminal might be increased in RF-EMF exposure group.

### The number of SVs is augmented at the calyx of Held presynaptic terminal in the auditory brainstem of P8 mice

**Figure 3:**
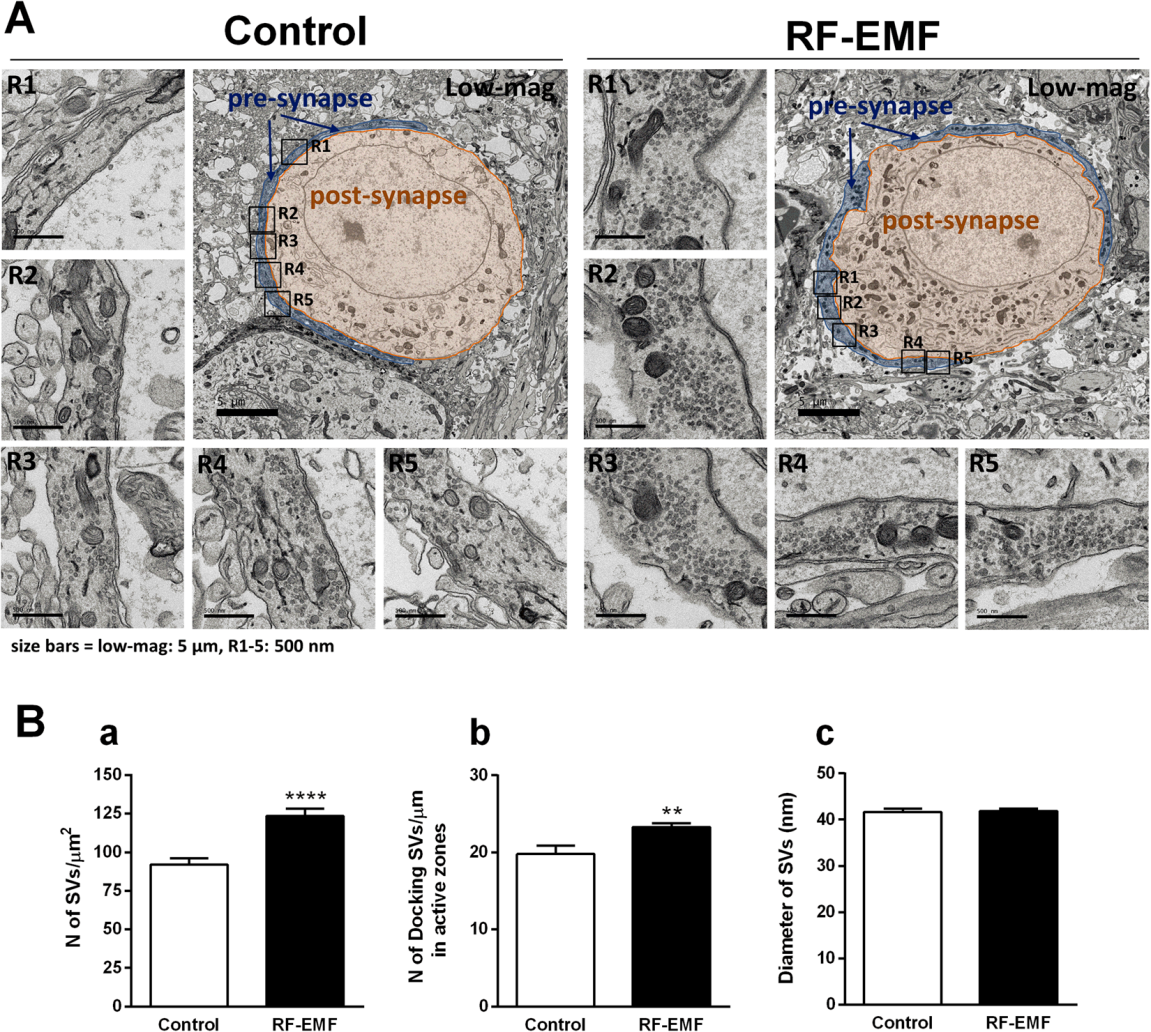
Ultrastructure of two MNTB neurons. (**A**) Transmission electron microscopy (TEM) image of a synapse at the medial nucleus of the trapezoid body (MNTB) of two mice at P8. Representative images of large excitatory synapses were acquired from a sham-exposed control mouse (left) and a RF-EMF exposed mouse (right). A single large calyx of Held nerve terminal (pre-synapse) and the MNTB principal cell (post-synapse) in the MNTB synapse are highlighted in blue and orange, respectively. Electron microscopy images at enhanced magnification (R1–5) show synaptic vesicles (SVs) and distinguishable active zones/ non-active zones. Scale bars, 5 μm (low-mag); 500 nm (R1–5). (**B**) (a) Number of synaptic vesicles (SVs) in the calyx of Held nerve terminals and docking SV number at the active zone in the membranes of presynaptic terminals. The number of SVs in the calyx of Held in the control group (*n* = 4) and RF-EMF exposed group (*n* = 4) were counted from 26 control and 29 RF-EMF exposed pre-synapses. The density of SVs was calculated as SV number/μm^2^. (b) The docking SVs at the active zones as well as the SVs within 100 nm from the active zones were counted (14 control and 15 RF pre-synapses). The total number of docking SVs at active zones was *n* = 2181 (control) and *n* = 3241 (RF-EMF exposed) vesicles, obtained from 80 (control) and 79 (RF-EMF exposed) enhanced images of the calyx of Held, originating from two different mice per group. On average, 5–6 images of enhanced magnification per calyx of Held were used. The density of docking SVs at the active zones was calculated. (c) The size of SVs (diameter; nm) at the presynaptic terminals in the calyx of Held between sham-control group (14 pre-synapses; 1984 SVs) and RF-EMF exposed group (15 pre-synapses; 2612 SVs). Each bar represents the mean ± SEM. Statistical significance was evaluated using a two-tailed unpaired Student *t*-test: **P < 0.01, ****P < 0.0001.

To determine whether the alteration of sPSCs in postsynaptic MNTB neurons after RF-EMF exposure was due to changes in the synaptic transmission at calyx of Held synapse, we counted the number of synaptic vesicles at the presynaptic terminal of the calyx of Held in P8 mice. We randomly selected and analyzed one ultrastructural image of the calyx of Held from each experimental group (Control n = 26, RF-EMF n = 29). Figure 3 shows a representative image of a calyx of Held synapse of a control (Fig. 3A) and an RF-EMF exposed (Fig. 3B) P8 mouse.

The number of SVs in calyx-type nerve terminals was counted in each condition (Fig. 3A. R1–5). We then calculated the total number of SVs in a unit area (μm^2^) of the calyx presynaptic terminals. The number of SVs in the unit area of the calyx presynaptic terminals was significantly higher in the RF-EMF group than in the control (123.4 ± 4.907 vs. 92.02 ± 4.164) (Fig. 3Ba). However, the size of the SVs (diameter; nm) did not differ significantly in the calyx presynaptic terminals after RF-EMF exposure (control; 41.65 ± 0.7712 and RF-EMF; 41.91 ± 0.5060) (Fig. 3Bc).

### The number of docking SVs is increased at active zones in the RF-EMF group

**Figure 4:**
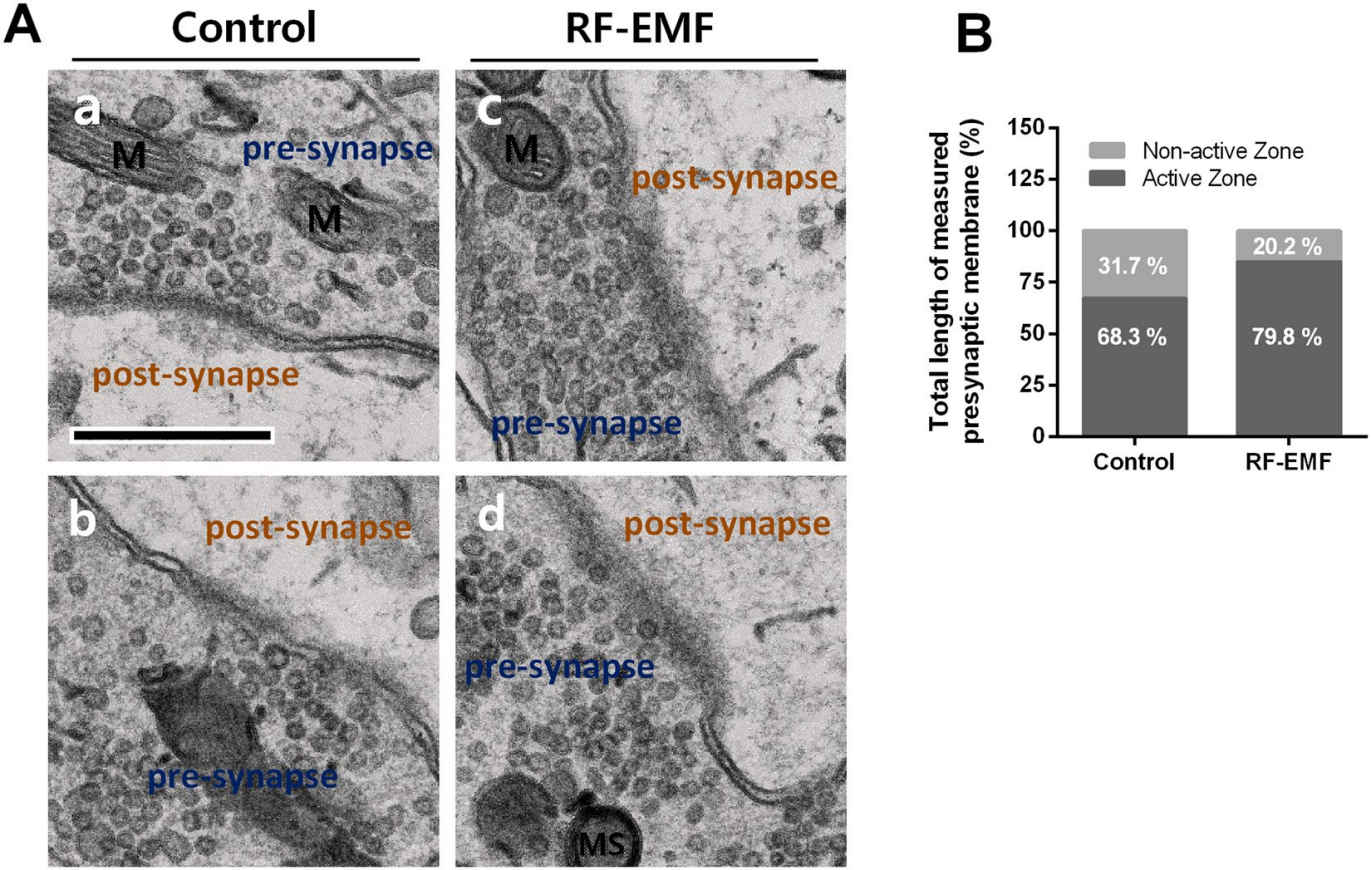
Ultrastructure of docking synaptic vesicles (SVs) at the active zones in the calyx of Held presynaptic nerve terminals. (**A**) Electron microscopy images at enhanced magnification of control mice (a,b) and RF-EMF exposed mice (c,d) show the active zones, which were formed by fusion of the SVs to the membranes. The blurry lines indicate the active zones in the presynaptic terminals. The total length of the active and non-active zone was measured from *n* = 80 and *n* = 79 enhanced images of calyx of Held originating from 2 control and 2 RF-EMF mice, respectively. On average, 5–6 images of enhanced magnification per calyx of Held were used. M, mitochondria; MS, myelin sheath. Size bars: 1 μm. (**B**) Total length of the presynaptic membrane of calyx of Held was estimated for the active zone and the non-active zone in control mice and RF-EMF exposed mice. Statistical significance in each group was evaluated using a two-tailed unpaired Student *t*-test and each was *P* < 0.0001.

As the actual transmission is determined by the number of docking SVs in the active zone, we counted the number and density (SVs numbers/μm) of docking SVs in the active zones of the calyx of Held presynaptic terminal membrane. The number of the docking SVs at the active zones was significantly larger in the RF-EMF exposed (23.28 ± 0.536), compared to the control (19.78 ± 1.113) group (Fig. 3Bb). In addition, we compared the ratio of the active zone to the non-active zone in the calyx presynaptic membranes. Representative TEM images are shown in Fig. 4A. In the control group, the total length of active zones was 105.4 μm (68.3%) and that of non-active zones was 48.8 μm. In the RF-EMF group, the total length of the active and non-active zones was 140.4 μm (79.8%) and 35.6 μm, respectively. Thus, the active zones were 12% longer in the RF-EMF group than in the control group (Fig. 4B). These results show that both the number of SVs and the length of the active zones at the presynaptic terminal were significantly larger in the RF-EMF group, suggesting that RF-EMF exposure enhances neurotransmission at calyx of Held synapses.

### Ribbon synapses in inner hair cells do not show morphological changes after RF-EMF exposure

**Figure 5:**
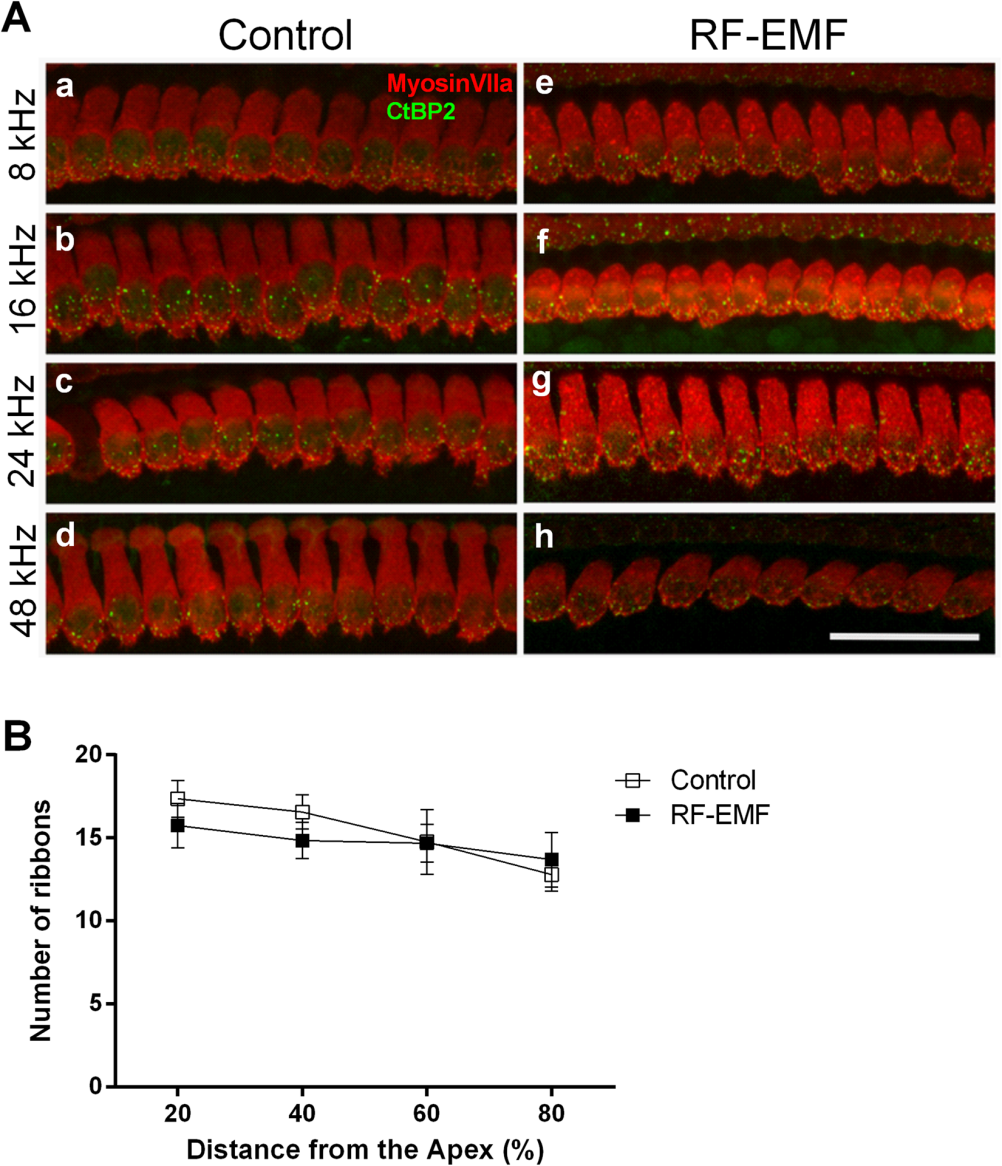
Status of ribbon synapse in the inner hair cell from both control and RF-EMF exposed groups. (**A**) Inner hair cell (IHC) and synaptic ribbons at four locations, representing 8 (a,e: 20% distanced from apex), 16 (b,f: 40% distanced from apex), 24 (c,g: 60% distanced from apex), and 48 (d,e: 80% distanced from apex) kHz in both control (a–d) and RF-EMF exposure groups (e–h) were selected for histological analysis. Ribbon synapses were located at the bottom of inner hair cell in both groups. Morphological or placement differences of ribbon synapse were not observed. Scale bar represents 40 μm. (**B**) Quantification of ribbon synapses in the IHC. Number of ribbons in IHC at four tested regions in both group were counted and were compared. Statistical analysis revealed no difference in all regions of the organ of Corti.

The above results suggested that the increased sPSCs amplitude and frequency might be due to a greater number of presynaptic vesicles. In turn, these increases might be a consequence of an elevated transmission at cochlear inner hair cell synapses. To explore this possibility, we counted the number of ribbon synapses at inner hair cells using immunohistochemistry. From the apex of the cochlea, we selected and compared four different regions, responding to sound frequencies of 8, 16, 24, and 48 kHz (Fig. 5A). We the counted the number of ribbon synapses located at the bottom of inner hair cells and compared them between the control and RF-EMF exposure group. In all tested regions, the number and morphology of hair cells were normal in both the control and exposure group. We found no significant differences between the groups (Fig. 5B).

### Auditory Brainstem Response (ABR) test

**Figure 6:**
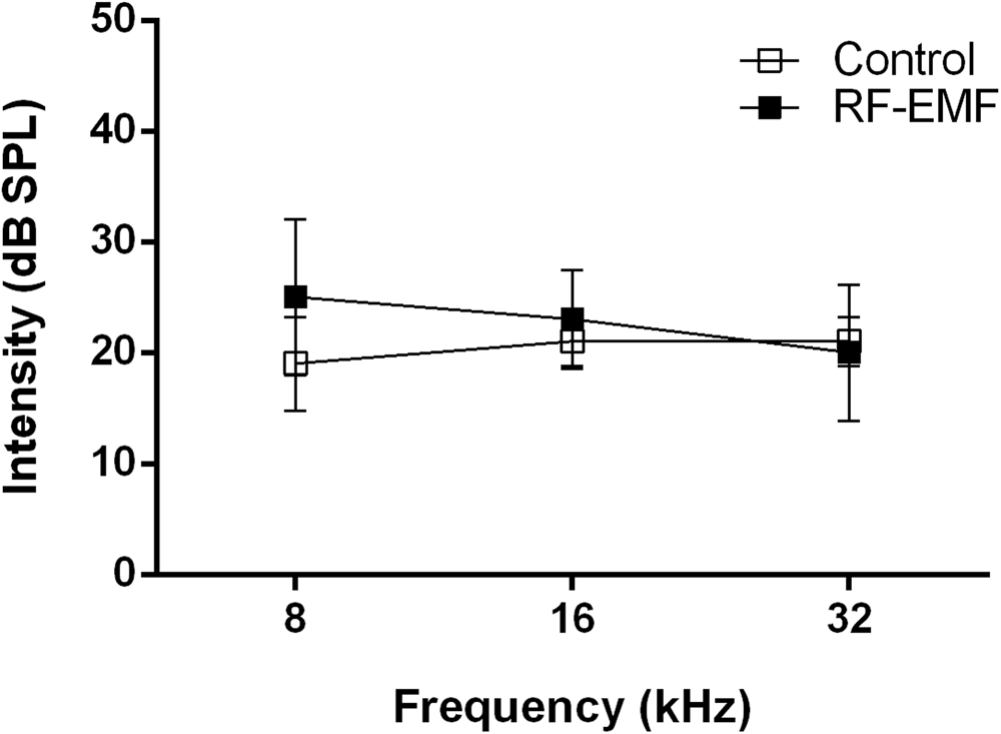
Auditory Brainstem Response (ABR) test from both control and RF-EMF exposed groups. The hearing threshold was measured using the auditory brainstem responses (ABRs) test from both control mice (*n* = 5) and RF-EMF exposed mice (*n* = 5) at P15. Tone stimuli with 8, 16, and 32 kHz were generated from 80 dB to 10 dB in 5 dB steps and average waveforms were generated from 1024 responses. There was no significant difference between the control and the RF-EMF exposed group (Student *t*-test, *P* < 0.05).

To explore the potential effects of RF-EMF exposure on the development of auditory functions in early postnatal mice, we tested the auditory brainstem response (ABR) at P15. There was no difference in ABR at any of the frequencies between control and RF-EMF exposed mice (Fig. 6). These results indicate that overall hearing was not affected, despite changes in neurotransmission at CN-MNTB synapses in the RF-EMF exposed group.

## Discussion

In this study, we found that 1850 MHz RF-EMF exposure increases the frequency and amplitude of sPSCs at post synaptic cells of the MNTB. Moreover, we found that the number of synaptic vesicles in the calyx of Held was significantly increased in presynaptic terminals. The number of docking synaptic vesicles at the active zone was increased by RF-EMF exposure and the total length of presynaptic active zones in the calyx of Held were also significantly increased. However, the number of ribbon synapses in the inner hair cells and the hearing threshold, as tested by the ABR test were not affected by RF-EMF exposure.

In early postnatal mice, the frequency and amplitude of sPSCs at postsynaptic cells of the MNTB were increased by the exposure to 1850 MHz RF-EMF. Because the MNTB is an auditory brainstem nucleus, any changes to MNTB neurons may affect auditory functions. Usually, mobile phones are generally held close to the ear and locally increases the exposure to RF-EMF. Therefore, there are high chances auditory brainstem to exposure to RF-EMF and the possible biological effects of RF-EMF exposure on neuronal activity within the auditory system should be studied more critically.

Recently, it has been reported that exposure to ELF-EMF facilitates vesicle endocytosis and synaptic plasticity in a calcium-dependent manner by increasing calcium channel expression at the MNTB^12^. These data provided a novel insight that ELF-EMF exposure can change neuronal activity by increasing the number of voltage-gated calcium channels. Calcium ion has an important function in regulating various neuronal processes including excitability, neurotransmitter release, and synaptic plasticity^15^. The postsynaptic principal neurons of the MNTB may be affected by alterations in the intracellular calcium concentration in presynaptic cells of the calyx of Held synapse due to RF-EMF exposure. However, our findings of increased sPSCs amplitudes under a low calcium and high magnesium condition suggest that other factors than enhanced calcium channel expression may be involved.

We found that the number of SVs in the presynaptic terminal was significantly increased in the RF-EMF group but the size of SVs was not changed. Neuronal signal transmission is directly affected by the number of SVs in the active zones in the calyx of Held presynaptic terminal membrane. Therefore, we measured the density of docking SVs in the active zones. The RF-EMF group showed a significant increase in the number of the docking SVs in the active zones. Because synaptic vesicles, located at the presynaptic nerve terminal, mainly regulate neurotransmitter storage, release, and secretion, numerical or morphological changes in SVs may cause alterations in synaptic transmission^16^. The total length of the presynaptic membrane of the calyx of Held was significantly increased in the RF-EMF group. These data suggested that more neurotransmitter would be released to synapses and contributed to increase the amplitude of sPSCs and the frequency of neurotransmitter release would be increased at the membrane of the calyx of Held in the group of RF-EMF exposure. It is possible that the increased number of SVs at the active zones and the longer presynaptic membrane at the calyx of Held led to an increased sPSC frequency and amplitude at MNTB postsynaptic neurons. Both the increase in the number of SVs and in the lengthening of active zones in calyx presynaptic terminals could directly increase the release of neurotransmitters. Specifically, the present study indicates that RF-EMF exposure during P1–P5 can increase electrical activity in postsynaptic principal cells of the MNTB due to highly active presynaptic transmission as a result of increased SVs and active zones. Although we have not shown the enhanced expression of VGCC in presynaptic terminal of principle cell, our results suggest that the increased proportion of active zone might indicate the increased number of VGCC in presynaptic terminal. Also, it is consistent with the numbers of presynaptic Ca^2+^ channel clusters match those of functionally defined vesicular docking sites in parallel fiber-molecular layer interneuron synapses^17^.

However, increased number of docking SVs and the elongated active zones might not explain the increased sPSCs completely. Compared to that of control group, the observed frequency of sPCSs is 2.5 times greater and the enhanced frequency was only observed in normal Ca^2+^ concentration condition, thus, some Ca^2+^ related factors should be considered. The exact mechanism of enhanced frequency of sPSCs was not suggested in the previous paper^12^. Their only suggestion is that increased expression of presynaptic VGCC might activate some calcium dependent process, such as the Protein kinase C pathway, and it might accelerate vesicle endocytosis and potentiate post-tetanic potentiation. This mechanism should be considered in our study, too. As PKC activation is well known factor enhancing sPSCs in the calyx of Held synapses and it is reported that PKC potentiates transmitter release by increasing the Ca^2+^ sensitivity of vesicle fusion^18^. In our study, the enhanced frequency of sPSCs of RF-EMF mice was only observed at normal external [Ca^2+^] condition. In low [Ca^2+^] and high [Mg^2+^] condition, in which calcium currents through VGCC might be very small, frequencies of sPSCs recorded from both groups were not statistically different. This result also might be in line with presynaptic calcium dependent process. Whether PKC is the true contributor for the enhanced sPSCs frequency in RF-EMF group needs to be studied in the future.

It is well known that changes in the inner ear affect auditory circuits in the brainstem. A previous study reported that a destruction of the developing inner ear by administration of kanamycin could induce changes in the neurotransmission at MNTB-LSO synapses^19^. Therefore, the increase in the number of sPSCs and SVs might have been caused by an increased activity of ribbon synapses in the inner hair cells. Because the ribbon synapse holds synaptic vesicles close to the active zone and is involved in the control of neurotransmitter release, auditory nerve activity is affected by the number of synaptic ribbons^20,21^. However, since we did not observe an increase in the number of ribbon synapses it is more likely that the increase in the number of sPSCs and SVs is a consequence of direct RF-EMF effects on the brainstem, rather than via inner ear. Our recent report showing that 835 MHz RF-EMF could induce changes in the number and size of synaptic vesicles in cortical neurons is also supportive to this hypothesis^5^. However, we did not observe any significant changes in the ABR test, suggesting that changes of sPSCs at calyx of Held synapses did not alter hearing threshold. Thus, our data suggest, that hearing sensitivity and peripheral sound perception are not affected by RF-EMF exposure. However, it may still be possible that RF-EMF exposure can affect sound localization abilities. This suggests that exposure to RF-EMF does not affect the peripheral hearing organ, i.e., the cochlea, but it may directly alter the number of SVs in the developing auditory synaptic circuit.

In summary, synapses in the auditory brainstem of early postnatal (P8) mice are affected by exposure to 1850 MHz RF-EMF (SAR of 4.0 W/kg for 5 h/day for 5 days), and show an increased sPSC frequency and amplitude, as well as a larger number of SVs in the active zone in the calyx of Held. RF-EMF exposure at an early postnatal stage can thus induce circuit changes in the auditory nervous system, without affecting functions of sound perception.

## Material and Methods

### Animals

ICR pups (postnatal day 0) with mother mouse were purchased from Samtako BioKorea (Osan, South Korea. The mice were maintained under specifically controlled conditions (ambient temperature 23 ± 2 °C, 12-h light/dark cycle). Pups were fed breast milk from their mother that was supplied food pellets and water ad libitum. All procedures complied with National Institutes of Health guidelines of the NIH for animal research and were approved by Dankook University Institutional Animal Care and Use Committee (IACUC; DKU-15-001), which adheres to the guidelines issued by the Institution of Laboratory of Animal Resources.

### RF-EMF exposure

Mice were exposed to electromagnetic field by using an 1850 MHz RF generator. We had tested and described about dosimetry for this RF generator in detail^3^. Firstly, we confirmed that RF-EMF generator created 1850 MHz signal by measuring spectrum analyzer (NS-30A) (LIG Nex, Gyeonggi-do, South Korea). RF signal and SAR value generated from our RF-EMF generator produce 1850 MHz RF-EMF with 4.0 W/kg SAR. Whole body exposure was at a SAR value of 4.0 W/kg for 5 hours daily for consecutive 5 days between 9:30 and 14:30. The other pups also received sham exposure for 5 days. The sham-exposed group was kept under the completely same environmental conditions and treated the same circular pattern as the RF-exposed groups without RF-EMF exposure. The sham-treated and RF-exposed mice could move freely in their cage. The bottom and wall of the cage were covered by ceramic wave absorption material. All the experiments have been done in our animal facility, which were maintained in constant temperature. The horn antenna for RF-EMF exposure is located at the top of the mouse cage. Pups with mother mouse were exposed whole body on 1850 MHz RF-EMF at a SAR value of 4.0 W/kg for 5 hours per day for consecutive 5 days (from P1 to P5) and had a resting period for 2 days. At p8, mice of either sex were sacrificed for electrophysiological or biochemical studies.

### Slice preparation for electrophysiology

After the mice received deep anesthesia with isoflurane, the brains were removed in ice-cold artificial cerebrospinal fluid (aCSF) and 300 μm thick slices containing the medial nucleus of trapezoid body (MNTB) were cut with a vibratome (LEICA VT1000s, LEICA Microsystems, Heidelberger, Germany). The slices were allowed to recover for at least 30 minutes in an interface chamber before recording. The slices were transferred to a submersion-type chamber mounted on an upright microscope and perfused continuously with aCSF containing (in mM) NaCl (124), KCl (5), KH_2_PO_4_ (1.25), MgSO_4_ (1), glucose (10), NaHCO_3_ (26), and CaCl_2_ (2). Electrophysiological recordings were made at room temperature.

### Electrophysiology

Whole cell patch clamp recordings were made at the postsynaptic cells of MNTB neurons. sPSCs were analyzed off-line using event detection threshold search mode provided by Clampfit 10.3. Every sPSC with amplitude greater than threshold was examined by eye for its acceptance as a true sPSC. The recording electrodes (3–5 MΩ) were filled with solution containing (in mM) K-gluconate (130), EGTA (1), HEPES (20), Na_2_GTP (0.3), K_2_ATP (1), KOH (11), KCl (4), and QX 314 (5 mM). All chemicals except QX 314, CNQX (Tocris Bioscience) were purchased from Sigma. In whole cell recording, except the fast transient cancelation, neither series resistance compensation nor whole cell capacitance cancelation was made. The data were filtered at 5 kHz (EPC-8, HEKA, Germany), digitized at 10 kHz, and stored on a computer using a homemade program (R-clamp 1.23). The analysis of the electrophysiological data and statistical testing were performed with Clampfit 10.3 (Molecular Devices) and Origin 7.0 (OriginLab Corporation). Data are expressed as mean ± standard error of the mean throughout the text.

### Transmission electron microscopy

Tissue samples were collected from 300 μm thick slices containing calyx of Held synapses in the medial nucleus of trapezoid body (MNTB) which were cut with a vibratome (LEICA VT1000s, LEICA Microsystems, Heidelberger, Germany). The brain slices were immediately fixed in 2% glutaraldehyde and 2% paraformaldehyde in 0.1 M phosphate buffer (pH 7.4) for 2 h at 4 °C. Following three washes in phosphate buffer, the brain tissues were post-fixed with 1% osmium tetroxide on ice for 2 h and washed three times, all in phosphate buffer. The tissues were then embedded in Epon 812 after dehydration in ethanol and propylene oxide series. Polymerization was conducted with pure resin at 70 °C for 24 h. Ultrathin sections (~70 nm) were obtained with a model MT-X ultramicrotome (RMC, Tucson, AZ) and collected on 100 mesh copper grids. After staining with uranyl acetate and lead citrate, the sections were visualized using Bio-HVEM System (JEM-1400 Plus at 120 kV and JEM-1000BEF at 1,000 kV (JEOL, Japan)).

### Measurements for number and size of SVs

Samples were prepared with control mice (n = 4) and RF-EMF exposed mice (n = 4). We generated images of 6–7 calyx of Held synapses in MNTB neuron (low-magnificent) per mouse and the images of calyx of Held excitatory nerve terminals (pre-synapse) were enlarged (enhanced magnification) to count the synaptic vesicles (SVs). We totally obtained 140 and 171 enhanced magnification images from 26 and 29 calyxes of Held synapses in control and RF-EMF exposed group, respectively.

In addition, we simply selected only the SV membrane which was clearly distinguished and then number of the synaptic vesicles (SVs) in all presynaptic area were counted (control 18,795 and RF-EMF 27,634 SVs). Total number of SVs per unit area (μm^2^) was calculated following the instructions of previous report^5^. The size of synaptic vesicle (SV) from 14 control pre-synapses (1984 SVs) and 15 RF pre-synapses (2612 SVs) was calculated by measuring the diameter (pixels) of each SV by drawing a line across the SV inner membrane using the ‘ImageJ’ program and the radius (pixels) of each SV was calculated (pixels). The pixel value was then calculated based on the scale bar to obtain the actual size value (nm).

### Counting the number of SVs at active zone

The docking vesicles in the active zone can be defined as a state in which the synaptic vesicle reaches at the presynaptic bouton and the SV membrane fuses with presynaptic terminal membrane^22^. The active zone is seen to be a dark and blurry area close to the presynaptic membrane^22^. To count the number of docking SVs at active zones, the enhanced magnification images of calyx of Held excitatory nerve terminals (pre-synapse) were used. The number of SVs per unit length (μm) was obtained following the instructions below. The number of pixels per 1 μm length was calculated by dividing the number of pixels of the acquired image by the length of the scale bar (5 μm) using Image J program. Active zone was considered to be the active zone only when the SV reached the membrane of the pre-synaptic terminal and fusion occurred between SV and membrane and thus, the membrane line appeared blurry. In addition, average diameter of 50 nm SVs located within 100 nm from the presynaptic terminal were counted. SV density was obtained by dividing total number of SV with the total length of the active zone.

### Auditory brainstem response (ABR) measurement

Auditory brainstem responses (ABRs) were measured to investigate the changes of hearing threshold before and after RF-EMF Exposure. The evoked response signal-processing system (System III; Rucker Davis Technologies, Alachua, Florida) was used for ABR measurement. Animals were anesthetized with zolazepam (Zoletil, Virbac, Carros Cedex, France) and xylazine (Rompun, Bayer, Leverkusen, Germany) and were placed in a soundproof chamber. Then, needle electrodes were inserted to the vertex (as response) and ventrolateral sides of both pinnae (as reference and ground). Tone stimuli with 8, 16, and 32 kHz were generated from 80 dB to 10 dB in 5 dB steps and average waveforms were generated from 1024 responses. The ABR test was done with p15 mice because the auditory system of mouse is immature at the time of birth and then they can hear around P12~P14.

### Immunohistochemistry

Animals were sacrificed after the last RF-EMF exposure and temporal bones were harvested. Samples were fixed in 10% Natural Buffer Formalin (NBF) for 2 hours at room temperature, rinsed with PBS, and the tissue containing the organ of Corti was dissected. Tissues were stained for MyosinVIIa to determine the inner and outer hair cell areas and with CtBP2 to evaluate the density of ribbon synapse. The dissected tissues were permeabilized and blocked in 0.3% Triton-100 with 5% Normal Goat Serum (NGS) for 30 minutes. Then, the samples were incubated with both primary antibodies (rabbit anti-MyosinVIIa, Proteus Biosciences #25-6790, 1:200 dilutions in 1% NGS and mouse anti-CtBP2, BD Transduction, #612044, 1:500 dilutions in 1% NGS) overnight. After washing with 1X PBS, tissues were incubated with the appropriate secondary antibodies, Alexa 488 and 568 (goat anti-mouse IgG1-488, Life Technologies #A21121, 1:1000 and Goat anti-rabbit IgG, abcam #ab175471, 1:1000) for two hours at room temperature.

Images were produced using a confocal microscope (FV-3000, Olympus, Japan). IHC synaptic areas were photographed in each sample at four frequency locations in the basal membrane (8, 16, 24, and 45 kHz, equivalent to 20, 40, 60, and 80% from the apex). Confocal z-stacks (0.5 um vertical spacing) were acquired with x63 oil-immersion objective and x2 digital zoom. Each stack included ~10 IHCs and all the ribbon synapses in each stack were counted and then divided according to the number of IHCs.

### Statistical analysis

The data are presented as the mean ± SEM. The n values represent the number of independent animal samples used in the experiments. Statistical analysis was made using the Student’s t-test unless otherwise stated and a p-value < 0.05 was considered statistically significant.
